# The Relationship between Personality Trait and Dental Anxiety in Students of Health-Related Specialties: A Pilot Study

**DOI:** 10.1055/s-0044-1791838

**Published:** 2024-12-10

**Authors:** Dania Al Khatib, Sereen Altaheri, Mera Ismail Al Sabh, Haydi Elshirbiny, Hiyam Adel Masaad, Tayebah AlAbdullah, Sarah Alsumait, Hanouf Alsulaili, Fatimah Buhamad, Natheer Hashim Al-Rawi

**Affiliations:** 1 Department of Oral and Craniofacial Health Sciences, College of Dental Medicine, University of Sharjah, Sharjah, United Arab Emirates; 2 Research Institute of Medical and Health Sciences, University of Sharjah, Sharjah, United Arab Emirates

**Keywords:** dental anxiety, personality traits, dental phobia, MDAS, Big Five

## Abstract

**Objectives** Dental anxiety is a common issue affecting a significant portion of the population, often leading to avoidance of dental care and subsequent oral health problems. Understanding the underlying factors contributing to dental anxiety is a crucial step toward developing an effective intervention. The aim of the study was to assess the prevalence of dental anxiety among students of health-related majors, evaluate their personality traits, and find the correlation between them. Additionally, this study aims to find the predictors of such traits and their relationship with each other.

**Materials and Methods** The study was conducted on 163 consented students (124 males and 39 females), selected based on major and year of study at the University of Sharjah medical campus. The questionnaire was sent online through Google Forms. It included questions from the Modified Dental Anxiety Scale (MDAS)and the Big Five personality test. Data analysis was done using SPSS software (IBM Co. version 29) where all descriptive and inferential statistics were conducted with statistical significance set at *p* < 0.05.

**Results** Moderate level of dental anxiety (12.3 ± 5.8) was observed among students of the medical campus, where nondental students scored higher in mean dental anxiety (13.0 ± 6.2) compared with dental students (10.4 ± 4.5). Males scored higher on the dental anxiety scale (12.5 ± 5.8) compared with females (11.7 ± 5.6). The Big Five personality test results displayed statistical significance association between neuroticism and dental anxiety, compared with other measured parameters ( *p* < 0.05).

**Conclusion** Neuroticism (Big Five characteristics scoring) and dental anxiety (MDAS scoring) exhibit a significant correlation. The Big Five test's characteristics are interrelated, including neuroticism and conscientiousness which, in turn, had a substantial correlation with agreeableness. Subsequently, agreeableness, conscientiousness, and extraversion exhibit substantial correlations with openness. This dynamic between the traits indicates that the adoption of personality tests in dental clinics would lead to improved prediction and management of dental anxiety in health-related students.

**Clinical Relevance** Since dentistry relies on patient management to get the best results, understanding the relationship between personality factors and dental anxiety might enhance patient management. This would prevent health care neglect and undiagnosed oral problems.

## Introduction

Anxiety disorders are becoming more prevalent, which has a substantial impact on daily life, such as the inability to access and receive the necessary health care. Anxiety disorders, including those associated with the health care milieu, are believed to affect 2.5 to 7% of the global populace. 1 2 Anxiety is a significant obstacle in the field of dentistry, as it is required that patients of all ages, from neonates to the elderly, undergo routine dental examinations. Worsened oral health outcomes are frequently the result of dental anxiety, which also contributes to poor oral hygiene and cycles of appointment avoidance. Anxiety is characterized by a sudden and continuous onset of intense restlessness and concern that culminates in minutes. It is frequently accompanied by physical symptoms, including increased heart rate, sweating, difficulty breathing, and difficulty sleeping. 3 4

Despite their familiarity with medical and/or dental settings and their understanding of medical and dental procedures, health care professionals are susceptible to dental anxiety. This familiarity does not shield them from experiencing dental anxiety. This may be the consequence of negative past experiences, cultural misconceptions, or an inherent quality. Although there are numerous explanations, health care professionals continue to struggle to comprehend the precise function of personality traits in dental anxiety. 3 Therefore, it is now possible to comprehend how health care providers may experience anxiety as a result of the treatments they typically administer when the roles swap. 5

The purpose of this study is to investigate the prevalence of dental anxiety in health-related students as evaluated by the Modified Dental Anxiety Scale (MDAS) and different personality traits determined by the Big Five personality test. The novelty of this study is in differentiating how particular personality traits correlate with dental anxiety and its expression in health-related students who are part of the profession and well adapted to the settings. This article completely investigates the five personality qualities (openness, conscientiousness, extraversion, agreeableness, and neuroticism), the Big Five personality test, evaluated in the context of dental anxiety.

By emphasizing the influence of particular personality traits on dental anxiety, this research endeavors to enhance our comprehension of the psychological etiology of dental anxiety if health care providers assume the role of the patient. Furthermore, recognizing the relationship can facilitate the integration of personality traits assessment into dental clinical practice, thereby improving patient management.

## Materials and Methods

### Study Design and Sample

This cross-sectional study comprised 163 consented students from the medical campus colleges (colleges of medicine, dentistry, pharmacy, nutrition, and health sciences). Each study participant gave informed consent. An informed consent form explained the study's purpose, procedures, risks, benefits, and participants' rights. Before participating in the study, participants signed consent forms. Informed consent was written at the beginning of the questionnaire. The questionnaire was distributed and answered using online Google Forms for 5 months (from November 2022 to March 2023). The questionnaire consisted of two tests (MDAS) to identify the dental anxiety levels of the participants with a benchmark of 19 which if passed would signal dental phobia and “The Big Five personality test” to identify the personality traits that treat personalities as a mix ratio of five main traits (openness, conscientiousness, extraversion, agreeableness, and neuroticism). The study was approved by the Institutional Review Board at the University of Sharjah with reference number (REC-22-11-20-01-S).

### Participant Selection Criteria

Female and male participants who majored in undergraduate health-related degrees are willing to participate voluntarily. The undergraduate health-related majors include medicine, dentistry, pharmacy, nutrition, and health sciences at any undergraduate study year (years 1–5). Since the survey was solely distributed on the medical campus, all participants belonged to health-related majors.

Participants are divided into “dental” and “nondental” majors. Any “uncategorized” answers were included in the nondental group since these are students who still have not yet decided on their major. Since these participants are “undecided,” it means that they have not been exposed to the dental education curriculum, thus considered “nondental.”

### Questionnaires: MDAS and Big Five

The MDAS consists of five questions presented to the participants in English. The test items explore the participant's feelings (extremely anxious to not anxious) before and during the day of the appointment. The range of answers corresponds with a score: extremely anxious = 5, very anxious = 4, fairly anxious = 3, slightly anxious = 2, and not anxious = 1. The sum of every answer's value is taken, and the total score is calculated at a 19-cutoff value. Scores at or above 19 were indicative of dental anxiety. 6 The MDAS was chosen for its widespread use and validation in assessing dental anxiety within the clinical setting. The choice of making 19 as the cutoff value has been proven by existing literature to be the score of highest sensitivity and specificity in clinical trials. 6 The Big Five is a highly reliable, popular, and clinically used 7 8 personality test consisting of 44 questions targeting five categories of traits presented in English. 9 The test claims that every human personality is a complex mix of five “big” traits with different strengths which are openness, conscientiousness, extraversion, agreeableness, and neuroticism. Thus, there is no cutoff score for this test, the higher the score, the stronger its presence in the individual's personality. The questions of the test target to explore the strength of this trait, and they are grouped in Table 1 . Each question item of the test corresponds to one of the five traits: openness, conscientiousness, extraversion, agreeableness, and neuroticism. The scores of the question of every trait are calculated using the formula as shown in Table 1 . Each number in the formula represents questions being asked in the survey (i.e., number 1 represents the first question of the survey test). The method of score calculation is explained in Table 1 . 10

**Table 1:** Scoring equations for the Big Five personality questionnaire

Big Five scale scoring (“R” denotes reverse-scored item)	
Extraversion	1, 6R. 11, 16, 21R, 26, 31R, 36
Agreeableness	2R, 7, 12R, 17, 22, 27R, 32, 37R, 42
Conscientiousness	3, 8R,13, 18R, 23R, 28, 33, 38, 43R
Neuroticism	4, 9R, 14, 19, 24R, 29, 34R, 39
Openness	5, 10, 15, 20, 25, 30, 35R, 40, 41R, 44
**Method of calculation** Every trait has a group of questions dedicated to it in the test. The numbers listed above are the numbers of the questions corresponding with the survey test (i.e., 1 corresponds with the first question in the survey test) and the scores 1–5 are given to each question depending on how much the test taker relates to it (1 = strongly disagree, 2 = disagree, 3 = neutral, 4 = agree, and 5 = strongly agree). Questions with “R” denote reverse, which means that it is a negative score, so it will be subtracted from the sum. The scores of all the questions in the group are added together (and any question with “R” should be subtracted as previously mentioned).	

### Statistical Analysis

All data analysis were conducted using SPSS version 29 (IBM SPSS for Mac, IBM Corp.). The analysis included means and standard deviations which were calculated for all the needed variables. Correlation tables were made to analyze the relationship between each of the Big Five personality traits with dental anxiety. A two-tailed *t* -test was used to analyze the demographic data in relation to dental anxiety. Similarly, a one-way analysis of variance (ANOVA) test was used to compare the average anxiety levels.

## Results

**Fig. 1:**
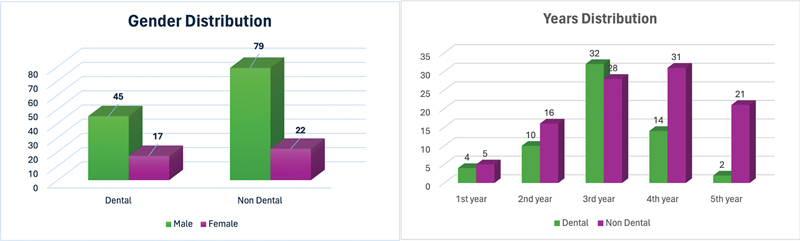
Demographic distribution of population.

The general characteristics of the studied population are depicted in Fig. 1 . To create a coherent flow in analyzing the demographics, *t* -tests, and ANOVA were used on four progressive levels. First, to get the inclusive dental anxiety score of the selected sample, a statistical analysis of dental anxiety among the entire sample was done as shown in Table 2 . It showed that the range of scores of the MDAS was from 5 to 25 with a mean score of 12.3 ± 5.8. This indicates a moderate level of dental anxiety among the sample.

**Table 2:** Statistical analysis of dental anxiety among the entire sample

Anxiety	
Sample number	163
Mean anxiety score	12.3
Median	11.0
Standard deviation	5.75
Range	20.0
Minimum	5.00
Maximum	25.0

Note: A mean score equal to or greater than 19 is indicative of dental anxiety.

Second, to get a more specific outlook on the dental anxiety of the participants, the sample was further divided based on the year of study. In Table 3 , a statistical analysis was done to compare the dental anxiety levels in the different year groups. Participants in year 4 scored highest in dental anxiety (13.7 ± 5.6) followed by year 5 (13.2 ± 5.9), year 2 (12.8 ± 5.8), year 1 (11.3 ± 4.3), and year 3 (10.8 ± 5.8), where *F* -value is equal to1.95 ( *p* > 0.05). This indicated that the most dentally anxious subgroup of the sample was year 4 students.

**Table 3:** Statistical analysis of anxiety among different study year groups

Class	Mean ± SD	*F* -value	Significance
Year 1	11.3 ± 4.3	1.95	0.10
Year 2	12.8 ± 5.8		
Year 3	10.8 ± 5.8		
Year 4	13.7 ± 5.6		
Year 5	13.2 ± 5.9		
Total	12.3 ± 5.7		

Abbreviation: SD, standard deviation.

The sample was then divided into two subgroups based on gender to further examine the dental anxiety levels among genders. A statistical analysis of dental anxiety among males and females is shown in Table 4 . The results of the analysis revealed that males had a higher score of dental anxiety (12.5 ± 5.8) compared with females (11.7 ± 5.6), where *F* -value is equal to 0.155 ( *p* < 0.05). Finally, the dental anxiety level and personality traits based on the study majors of the sample were examined. The sample was divided into “dental” and “nondental” majors. In Table 5 , an independent *t* -test was made to compare the group statistics between dental and nondental students' personality traits and dental anxiety. The personality traits' comparison between the two groups was not significant ( *p* > 0.05). However, the dental anxiety and dental phobia results showed significant differences ( *p* < 0.05). The results showed higher levels of anxiety in nondental students compared with dental students, 13.0 ± 6.2 and 10.4 ± 4.5, respectively. Another significant result was that the dental group showed more likeliness of dental phobia than the nondental group, 1.95 ± 0.2 versus 1.80 ± 0.4, respectively. The results in Table 5 provide a guide to further examine the association of personality traits and anxiety together. Therefore, a correlation table was made as shown in Table 6 . The correlation table compared the Big Five personality traits (openness, conscientiousness, extraversion, agreeableness, and neuroticism) to the score of dental anxiety. The results of the correlation table indicated that there is a significant positive correlation between dental anxiety and neuroticism ( *r* = 0.314, *p* < 0.01). There were no significant correlations between dental anxiety and the other traits. These findings suggest that certain personality traits may be more strongly related to dental anxiety than others. The correlation table further unravels the depth of neuroticism and its connection with the remaining traits. Initially, a significant negative correlation between neuroticism and conscientiousness was obvious where the correlation coefficient *r* = −0.157 ( *p* < 0.05) and with extraversion *r* = −0.278 ( *p* < 0.01). In return, conscientiousness has a significant positive correlation with agreeableness where *r* = 0.344 ( *p* < 0.05). Next, openness has significant positive correlations with agreeableness where the correlation coefficient *r* = 0.189 ( *p* < 0.05), conscientiousness *r* = 0.240 ( *p* < 0.01), and extraversion *r* = 0.237 ( *p* < 0.01). This chain of associations highlights that all personality traits are interrelated and progressively lead to strengthening neuroticism. This means that individuals with higher neuroticism, low extraversion, and low conscientiousness are more likely to exhibit dental anxiety.

**Table 4:** Statistical analysis of anxiety among gender groups

**Anxiety**	**Gender**	***N***	**Mean ± SD**	***t*** **-Value**	***F*** **-value**
	Male	124	12.5 ± 5.8	0.757	0.155
	Female	39	11.7 ± 5.6		

Abbreviation: SD, standard deviation.

**Table 5:** Comparison between dental and nondental majors' personality traits, anxiety, and phobia

Personality trait	Major	*N*	Mean ± SD	*t* -Value	*p* -Value
Extraversion	Dental	62	5.80 ± 5.31	−1.65	0.28
	Nondental	84	7.34 ± 5.72		
Agreeableness	Dental	62	11.64 ± 3.99	−1.09	0.33
	Nondental	84	12.39 ± 4.15		
Conscientiousness	Dental	62	6.77 ± 4.31	−0.10	0.12
	Nondental	84	6.85 ± 5.15		
Neuroticism	Dental	62	9.03 ± 5.71	0.48	0.78
	Nondental	84	8.57 ± 5.74		
Openness	Dental	62	23.41 ± 4.09	0.328	0.45
	Nondental	84	23.64 ± 4.04		
Anxiety	Dental	62	10.41 ± 4.47	−2.76	0.002 a
	Nondental	84	12.97 ± 6.18		
Phobia status	Dental	62	1.95 ± 0.216	2.72	< 0.001 a
	Nondental	84	1.79 ± 0.404		

Abbreviation: SD, standard deviation.

a Significant at *p* < 0.05.

**Table 6:** Correlation table comparing the Big Five personality traits to the score of dental anxiety

Trait	Extraversion	Agreeableness	Conscientiousness	Neuroticism	Openness	Anxiety
Extraversion	1					
Agreeableness	0.149	1				
Conscientiousness	0.122	0.344 a	1			
Neuroticism	−0.278 a	−0.14	−0.157 b	1		
Openness	0.237 a	0.189 b	0.240 a	0.102	1	
Anxiety	−0.045	−0.036	−0.111	0.314 a	0.119	1

a Significant at *p* < 0.01.

b Significant at *p* < 0.05.

To support the findings of Table 6 , a multiple regression analysis was conducted as shown in Table 7 . The standardized coefficient β represents the regression coefficients which show the magnitude and direction of the predictive relationship between personality traits and dental anxiety. As seen in Table 7 , neuroticism has the highest positive β value (β = 0.296) between all the independent variables, this means that whenever there is an increase in the level of neuroticism, the level of dental anxiety experienced increases. On the other hand, conscientiousness has a negative β value, −0.116, *p* < 0.01, this indicates that when the level of conscientiousness increases, the amount of dental anxiety decreases, and that supports the results of the correlation in Table 6 . This is also indicated by the unstandardized coefficient β which shows that among the five personality traits, neuroticism has the greatest effect on anxiety (β = 0.297). Furthermore, looking at the *p* -values of each of the personality traits, we will see that neuroticism has the most significant effect on dental anxiety with a score of *p* < 0.001.

**Table 7:** Regression analysis shows dental anxiety as the dependent variable, personality traits as the independent variable, and the Big Five personality as well as the MDAS test which were used to determine the anxiety levels and personality traits, respectively

Coefficients a					
Model	Unstandardized *B*	Coefficients standard error	Standardized coefficients beta	*t*	Significance
1 (constant)	6.653	2.67		2.491	0.014
Extraversion	0.023	0.086	0.022	0.269	0.789
Agreeableness	0.023	0.113	0.017	0.204	0.839
Conscientiousness	−0.116	0.097	−0.098	−1.188	0.237
Neuroticism	0.297	0.081	0.296	3.657	< 0.001
Openness	0.144	0.113	0.104	1.278	0.203

Abbreviation: MDAS, Modified Dental Anxiety Scale.

a Dependent variable: anxiety.

The regression formula ( *ŷ* = *b* 0 + *b* 1 *x* ) was used to find the constant in Table 7 which is represented by *b* 0. 11 The value of the constant plays an important role in the formulation of the regression line as it represents the value that would be predicted for the dependent variable (dental anxiety) if all the independent variables were to equal 0 at once. Coefficient *Y* represents the dependent variable, while *x* represents the independent variable and *b* 1 represents the regression coefficient. The standardized coefficient β was calculated using the following formula: β = *b* (SD *x* /SD *y* ). 11 Once calculated, the standardized coefficient β is used for selecting variables as well as explaining the importance of them and the impact of changing one variable.

## Discussion

The objective of this investigation was to investigate the correlation between dental anxiety and personality traits in students of health-related disciplines. The study specifically examined the relationship between specific personality traits from The Big Five test and higher levels of dental anxiety scores on the MDAS. The data underwent a series of analytical tests, which resulted in both significant and insignificant findings, following a progressive investigation. The findings demonstrated a significant relationship between personality traits, specifically neuroticism and dental anxiety, in health-related students. Additionally, there is a correlation between the five personality traits. Furthermore, it demonstrates that the prognosis and treatment course can be significantly impacted by the prediction of the medical professional's behavior as a patient, which is derived from the examination of their personality characteristics.

The statistical tests' results were inherently skewed by the unequal ratio of males to females in this study, which demonstrated that males exhibited a higher level of dental anxiety. In contrast, Arkkila et al demonstrated that females are more susceptible to dental anxiety than their male counterparts. 12 The study years of the participants were also distributed irregularly. Consequently, the prevalence of dental anxiety about personality characteristics cannot be ascertained by this study in terms of whether gender and the year of study are influencing factors.

The findings of this investigation demonstrated that the personality trait “neuroticism” has a significant relationship with dental anxiety, in contrast to the other traits, which did not exhibit a strong correlation ( *r* = 0.314, *p* < 0.01). Another study discovered a slight positive correlation between neuroticism and dental anxiety; however, a stronger correlation was observed between genetic and environmental triggers. 13 Additionally, the self-consciousness scale revealed a correlation between dental anxiety and self-consciousness. However, the relationship between social anxiety and public self-consciousness was more robust.

Multivariate regression models identified neuroticism as the sole variable associated with dental anxiety, while feelings of loneliness were only positively correlated with general anxiety. Conversely, Aarabi et al observed no correlation between neuroticism and agreeableness and dental anxiety in their investigation. However, they did identify a correlation between extraversion and conscientiousness and anxiety. 14 Additionally, patients who experienced acute dental problems were more likely to experience dental anxiety than those who underwent routine dental examinations. Valdes-Stauber and Hummel (2021) agreed with this finding. 15 Arkkila et al observed a positive correlation between extraversion and dental anxiety, particularly in women, in contrast to our research. 12 Vassend et al performed a twin cohort study to evaluate the relationship between neuroticism, dental anxiety, and dental care–related pain, as well as the genetic and environmental influences on these phenotypes. They demonstrated that neuroticism is associated with dental anxiety and discomfort through both genetic and individual-specific environmental pathways, albeit to a lesser extent. 16

The results of this study are inconclusive in that they cannot verify that academic stress is not a contributing factor to dental anxiety, as the mean anxiety results were consistent across all years of study. Therefore, additional research is required to determine whether academic stress is correlated with dental anxiety or if it influences the number of dental visits. The findings of the study indicated that the behavior of the patient and their levels of dental anxiety can be significantly influenced by poor knowledge of the procedure, as nondental students exhibited higher levels of mean anxiety (12.97 ± 6.18) than dental students (10.41 ± 4.47). Academic stress is not the sole external factor. 17 Dental students are also well integrated into the clinic environment in comparison to nondental students. Additionally, the dental group demonstrated a higher likelihood of anxiety than the nondental group. 18 19 This raises the question of whether the levels of anxiety are influenced by the exposure to the health care setting as a health care provider.

Although the MDAS is a dependable and extensively used instrument for evaluating dental anxiety, it is insufficiently specific in distinguishing between “state” and “trait” anxiety. The limitation that this dual assessment implies is underscored by a recent systematic review. 20 This investigation emphasizes the critical importance of distinguishing between these constructs to improve their clinical relevance.

It is worth noting that the purpose of establishing this correlation between personality traits and dental anxiety is not to diagnose dental anxiety or dental phobia, but rather to enhance the prognosis of the case. The clinical implications of this correlation include the enhancement of patient management by predicting the prospective emotional state of the patient in relation to dental care. Additionally, the dentist's communication approach and persuasiveness in oral care education will be enhanced by their understanding of their patient's personality. 19 The main goal was to enhance the patient's oral health by creating a personalized treatment plan and taking into account their personality characteristics. Al Jasser et al conducted comparable research in 2019, which revealed that dental students exhibited the lowest anxiety levels among other health-related majors. 21 They attributed their findings to their comprehensive understanding of dental procedures and oral health. Nevertheless, this may be accurate if their anxiety was a result of a lack of knowledge, or in other words, “fear of the unknown.” Conversely, their anxiety may be further exacerbated as they become more knowledgeable and more engrossed in the field; this is where the phrase “ignorance is joy” becomes applicable.

Our results demonstrate the irony that dental students may experience dental anxiety, which may be associated with the gradual accumulation of anxiety levels. It is possible that the dental student experienced some degree of dental anxiety prior to enrolling in dental school, and their anxiety may have been exacerbated as a result of consistent exposure to the dental environment. However, it is imperative to address the critical inquiries of the onset of the anxiety, the triggers, and the indications of improvement or aggravation since the commencement of dental school to conduct future research. Furthermore, anxiety may be exacerbated by other factors, such as recent examinations or past traumatic experiences.

In health-related professionals, dental anxiety can result in an imbalance in their decision-making. When dentists, for instance, demonstrate dental anxiety, their personal bias may impede the quality of care and treatment they provide to their patients. The integration of personality assessments into dental education and practice enables educators and clinicians to more effectively identify and address anxiety among students and practitioners. Consequently, this enhances their preclinical training and decision-making. In addition to improving the well-being of health-related students, this method will also improve the quality of their treatment and performance as future health professionals.

The implications of this research are broad and require an open-minded and innovative appreciation. This is because the psychological and emotional approaches are often given less focus and importance in dental school programs. Such communication skills can only be gained with time and experience. So, the revelations of this research are proof that having a better understanding of patient personality is crucial to providing better dental care.

## Limitations

The generalizability of this study is restricted to university students from the medical campus, which is one of its limitations. To obtain more representative and robust results, it is necessary to employ a more diverse and extensive sample. Furthermore, to mitigate the confounding impact of academic stress, future research should encompass a more extensive age range. In addition, the conclusion must be more reliable and effective if the female-to-male ratio is adequately balanced.

Privacy concerns during the investigation impeded the acquisition of comprehensive data, including socioeconomic data, social status, living conditions, and prior traumatic experiences. Socioeconomic data, such as monthly income, can impact financial status and, as a result, the decision to seek dental care. Social status and living arrangements (either with parents or independently) are likely to induce guardian pressure to obtain dental care. Future studies on this subject would also be more effective and reliable if data on past traumatic experiences related to the dental scene were available. Furthermore, it is imperative to implement more exhaustive and cohesive assessment instruments to assess dental anxiety levels. It is imperative to conduct additional research in the field of psychology to gain a more comprehensive understanding of the intricacies of the human mind and personality. Additionally, future research must include anxiety assessments that clearly distinguish between state and trait anxiety, as well as fundamental personality traits. To improve future research, it is necessary to employ a diagnostic instrument based on the Diagnostic and Statistical Manual of Mental Disorders (DSM) to investigate the relationship between dental anxiety and specific phobia.

## Conclusion

This research indicates that dental anxiety is not limited to patients who are unfamiliar with the dental environment; it also impacts health-related students, including those in dental disciplines, regardless of their clinical knowledge and experience. The results indicate a substantial correlation between specific personality traits, particularly neuroticism. Furthermore, there are correlations between neuroticism, conscientiousness, and agreeableness. The findings of the investigation may contribute to a deeper understanding of the underlying causes of dental anxiety among students pursuing health-related fields.

## Recommendation

Further research is necessary to establish a definitive correlation between gender and dental anxiety, as well as to determine whether dental anxiety is influenced by the socioeconomic status of patients, age, and their own prior traumatic experiences, as well as the influence of witnessing others share negative thoughts.
